# Modulation of Cell Death and Promotion of Chondrogenic Differentiation by Fas/FasL in Human Dental Pulp Stem Cells (hDPSCs)

**DOI:** 10.3389/fcell.2020.00279

**Published:** 2020-05-15

**Authors:** Alessandra Pisciotta, Giulia Bertani, Laura Bertoni, Rosanna Di Tinco, Sara De Biasi, Antonio Vallarola, Elisa Pignatti, Rossella Tupler, Carlo Salvarani, Anto de Pol, Gianluca Carnevale

**Affiliations:** ^1^Department of Surgery, Medicine Dentistry and Morphological Sciences with Interest in Transplant, University of Modena and Reggio Emilia, Modena, Italy; ^2^Department of Medical and Surgical Sciences for Children and Adults, University of Modena and Reggio Emilia, Modena, Italy; ^3^Department of Biomedical, Metabolic and Neural Sciences, Center for Neuroscience and Neurotechnology, University of Modena and Reggio Emilia, Modena, Italy; ^4^Rheumatology Unit, Azienda Unitá Sanitaria Locale-IRCCS di Reggio Emilia, Reggio Emilia, Italy

**Keywords:** hDPSCs, Fas/FasL pathway, chondrogenic differentiation, 3D pellet culture, pericytes

## Abstract

Human dental pulp stem cells (hDPSCs) are characterized by high proliferation rate, the multi-differentiation ability and, notably, low immunogenicity and immunomodulatory properties exerted through different mechanisms including Fas/FasL pathway. Despite their multipotency, hDPSCs require particular conditions to achieve chondrogenic differentiation. This might be due to the perivascular localization and the expression of angiogenic marker under standard culture conditions. FasL stimulation was able to promote the early induction of chondrogenic commitment and to lead the differentiation at later times. Interestingly, the expression of angiogenic marker was reduced by FasL stimulation without activating the extrinsic apoptotic pathway in standard culture conditions. In conclusion, these findings highlight the peculiar embryological origin of hDPSCs and provide further insights on their biological properties. Therefore, Fas/FasL pathway not only is involved in determining the immunomodulatory properties, but also is implicated in supporting the chondrogenic commitment of hDPSCs.

## Introduction

Human dental pulp stem cells (hDPSCs) are located in the perivascular area of the loose connective tissue enclosed within the pulp chamber. Human DPSCs are characterized by a low-invasive procedures required for isolation, high proliferation rate, low immunogenicity and the ability to differentiate into different cell lineages (Gronthos et al., 2002). The multi/pluripotency of hDPSCs can be attributed to their particular embryological derivation from the neural crest. Particularly, neural crest cells originate during the formation of neural tube, at the third week of embryo development, then undergo an epithelial-mesenchymal transition (EMT) and migrate to different body compartments under the control of several regulatory factors. Following migration, neural crest cells generate the majority of craniofacial tissues, including tooth, fat, muscle, bone and cartilage tissues, as well as cranial peripheral ganglia and nerves, among other cell types, such as melanocytes (Pisciotta et al., 2020). It has been widely demonstrated that hDPSCs are able to differentiate toward lineages belonging to all the three germ layers, as a matter of fact these stem cells can commit to glial cells and participate in peripheral nerve regeneration (Carnevale et al., 2018), contribute in restoring urethral sphincter contractile function (Zordani et al., 2019), reduce fibrosis and ameliorate muscle trophism in Duchenne Muscular Dystrophy mouse model (Pisciotta et al., 2015b), besides promoting bone tissue regeneration in critical size calvarial defects (Pisciotta et al., 2012). Further evidence of the regenerative potential of hDPSCs is documented by their capability to promote vascularization in regenerating tissues *in vivo* and supported by their expression of VEGF (Laino et al., 2005).

On the other hand, the ability of hDPSCs to commit into chondrogenic lineage is debated and controversial. Indeed, findings from literature demonstrated that hDPSCs show high variability when induced toward chondrogenic differentiation: this is likely due to the heterogeneity of dental pulp cells (Iohara et al., 2006; Zhang et al., 2006) and to high oxygen levels when differentiating hDPSCs *in vitro*, that do not reflect the physiological *in vivo* oxygen tension required for articular chondrocyte differentiation (Chen et al., 2015). The only cartilage portion present in the craniofacial district is represented by Meckel’s cartilage, hyaline cartilage formed in the mandibular process of the first branchial arch of vertebrate embryos. From an embryological point of view, chondrocytes are differentiated from mesodermal cells in general, whereas cells forming Meckel’s cartilage, are differentiated from ectodermal mesenchymal cells of neural crest origin (Amano et al., 2010). Moreover, during intramembranous ossification cartilage is not present and MSCs from neural crest differentiate directly into osteoblasts.

Beside their differentiation potential, it has been widely demonstrated by several studies conducted *in vitro* and *in vivo* that hDPSCs can modulate the immune response through different mechanisms (Wada et al., 2009; Zhao et al., 2012). To this regard, it is renown that FasL is expressed in different cell types residing in “immune-privileged” sites, such as the testis, the eye and the nervous system (Brunlid et al., 2007). Therefore, it would be helpful to understand how FasL expression modulates hDPSCs properties.

Taken together, these properties represent the necessary criteria to define hDPSCs as a suitable stem cells source for regenerative medicine.

Several findings in literature have highlighted the prominent role of Fas/FasL pathway in immunomodulation. Particularly, the activation of Fas/FasL pathway occurs following the exposure to inflammatory microenvironment which induces apoptosis in T cells (Pierdomenico et al., 2005; Zhao et al., 2012; Riccio et al., 2014). Mechanistically, upon binding of FasL to its receptor CD95, or Fas receptor, the extrinsic apoptotic pathway is activated with Pro-Caspase 8 and Fas associated death domain (FADD) being recruited to form the death inducing signaling complex (DISC), in which Pro-Caspase 8 undergoes activation. Then, Caspase 8 leaves the DISC, activates caspase 3/7 and induces apoptosis (Chang et al., 2002; Carnevale et al., 2017). Alternatively, c-FLIP, a protease-deficient caspase homolog can interact with FADD and act as an apoptosis inhibitor (Irmler et al., 1997). Besides its key role in immune system homeostasis maintenance and prevention of autoimmunity, increasing evidence showed the involvement of Fas/FasL signaling in further cellular responses, such as inflammation, proliferation and regeneration (Chang et al., 2002). These data were further confirmed by previous findings demonstrating that hDPSCs do express Fas receptor under standard culture conditions (Pisciotta et al., 2018).

To this regard, the aim of our study was to investigate whether and how Fas/FasL pathway can affect the stemness features of hDPSCs and, particularly, the modulation of their chondrogenic potential.

## Materials and Methods

### Isolation of STRO-1^+^/c-Kit^+^ Human Dental Pulp Stem Cells and Immunophenotype Characterization

The study was conducted in accordance with the recommendations of Comitato Etico Provinciale-Azienda Ospedaliero-Universitaria di Modena (Modena, Italy), which provided the approval of the protocol (ref. number 3299/CE; 5 September 2017). Human DPSCs were isolated from third molars of adult subjects (*n* = 3; 18–25 years) undergoing routine dental extraction. All subjects gave written informed consent in compliance with the Declaration of Helsinki.

Cells were isolated from human dental pulp as previously described (Bianchi et al., 2017). Briefly, dental pulp was harvested from the teeth and enzymatic digestion was carried out through a digestive solution (3 mg/ml type I collagenase plus 4 mg/ml dispase in α-MEM). Pulp was then filtered onto 100 μm Falcon Cell Strainers, in order to obtain a cell suspension. Then, cell suspension was plated in 25 cm^2^ culture flasks and expanded in standard culture medium (α-MEM supplemented with 10% heat inactivated foetal bovine serum (FBS), 2 mM L-glutamine, 100 U/ml penicillin, 100 μg/ml streptomycin; all from Sigma Aldrich, St. Louis, MO, United States) at 37°C and 5% CO_2_. Following cell expansion, hDPSCs underwent immune-selection by using MACS^®^ separation kit, according to manufacturer’ instructions. Two sequential immune-selections were performed by using mouse IgM anti-STRO-1 and rabbit IgG anti-c-Kit primary antibodies (Santa Cruz Biotechnology, Dallas, TX, United States). The following magnetically labeled secondary antibodies were used: anti-mouse IgM and anti-rabbit IgG (Miltenyi Biotec, Bergisch Gladbach, Germany). The selection of a homogeneous hDPSCs population expressing STRO-1 and c-Kit was then evaluated by immunofluorescence analysis, as described below.

With the aim to provide a visual representation as to the location and degree of overlap between two wavelengths corresponding of STRO-1 and c-Kit, co-localization map was performed by NIS software as previously described (Gibellini et al., 2012). Briefly, the two-dimensional scatter plot diagram of the image was analyzed to evaluate the spatial colocalization of the signals and the Pearson’s correlation was calculated. Moreover, areas with the strongest colocalized signals, corresponding to pixels for both detectors, were selected to generate colocalization binary maps. In order to evaluate the expression of the typical mesenchymal stem cells (MSCs) markers, immune-selected hDPSCs at passage 1 underwent FACS analysis against CD73, CD90, CD105, CD34, CD45, HLA-DR, as formerly described by Conserva et al. (2019). Following trypsin dissociation, cells were resuspended in culture medium and were stained with the following fluorochrome-conjugated antibodies (Abs): anti-human-CD73-PE-CY7, -CD90-FITC, -CD105-APC, -CD45-PE, and -HLA-DR-PE-CY7 (all from BD Biosciences, Franklin Lakes, NJ, United States); and -CD34-ECD (Beckman Coulter, Fullerton, CA, United States). A minimum of 10,000 cells per sample was acquired and analyzed by using the Attune Acoustic Focusing Flow Cytometer (Attune NxT, Thermo Fisher, Waltham, MA, United States). Data were analyzed by FlowJo 9.5.7 (Treestar, Inc., Ashland, OR, United States) under MacOS 10.

STRO-1^+^/c-Kit^+^ hDPSCs were also analyzed for the expression of HLA-ABC and HLA-DP-DQ-DR by confocal immunofluorescence analysis. Cells were fixed with 4% paraformaldehyde (PFA) in pH 7.4 phosphate buffer saline (PBS) for 20 min and washed in PBS. After rinsing with PBS, samples were blocked with 3% BSA in PBS for 30 min at room temperature and then incubated with FITC-conjugated mouse anti-HLA ABC and mouse anti-HLA-DP-DQ-DR primary antibodies (BD Biosciences, Franklin Lakes, NJ, United States), diluted 1:50 in PBS containing 3% BSA, for 1 h at room temperature. Then, cells were rinsed thrice with PBS and finally, nuclei were stained with 1 μg/mL 4,6-diamidino-2-phenylindole (DAPI) in PBS for 5 min (Bianchi et al., 2017); then, samples were mounted with anti-fading medium (FluoroMount, Sigma Aldrich, St. Louis, MO, United States) and were observed by a Nikon A1 confocal laser scanning microscope. The confocal serial sections were processed with ImageJ software to obtain three-dimensional projections, and image rendering was performed using Adobe Photoshop Software (Carnevale et al., 2018).

### PBMCs Isolation and Co-culture With hDPSCs

Human peripheral blood was collected from healthy donors who gave written informed consent, according to the guidelines of the ethics committee. Peripheral blood mononuclear cells (PBMCs) were isolated by using Histopaque^®^ (Sigma Aldrich, St. Louis, MO, United States), according to the manufacturer’ instructions, and re-suspended at a density of 10^6^ cells/ml in RPMI 1640 medium (GIBCO^®^ Life Technologies Italy, Monza) supplemented with 10% FBS, 2 mM glutamine, 100 units/ml penicillin, and 100 μg/ml streptomycin (all from Sigma-Aldrich, St. Louis, MO, United States). PBMCs were pre-activated by adding anti-CD3 and the costimulatory anti-CD28 monoclonal Abs (1 μg/10^6^ PBMCs; BD Biosciences, Franklin Lakes, NJ, United States) to culture medium and then used for co-culture experiments (Carnevale et al., 2017). Briefly, for co-culture experiments hDPSCs were seeded in 6-well plates at a cell density of 7,000 cells/cm^2^ and cultured in RPMI 1640 medium supplemented with 10% FBS, 2 mM glutamine, 100 units/ml penicillin and 100 μg/ml streptomycin. Upon hDPSCs adhesion, after 24 h, pre-activated PBMCs were seeded on hDPSCs at a 5:1 ratio and kept in culture for 48 h. At the end of the direct co-culture, the floating PBMCs were removed together with the supernatant and discarded, whereas the attached hDPSCs were processed according to the subsequent experimental procedures.

hDPSCs and pre-activated PBMCs cultured alone were used as controls.

### hDPSCs-PBMCs Co-culture: Evaluation of Fas/FasL Pathway

In order to evaluate Fas/FasL pathway in hDPSCs cultured alone and after co-culture with PBMCs, the expression of Fas, FasL, Caspase 8 and c-FLIP, was investigated by Western blot analysis. Particularly, whole cell lysates were obtained as formerly reported (Pisciotta et al., 2018). Briefly, 30 μg of protein extract per specimen were quantified by a Bradford Protein Assay (Bio-Rad), then SDS-polyacrylamide gel electrophoresis and subsequent protein transfer to nitrocellulose membranes were performed. The following antibodies were used: rabbit anti-FasL, mouse anti-Fas, mouse anti-Caspase 8 (Cell Signaling Technology, Trask Lane Danvers, MA, United States), rabbit anti-c-FLIP (R&D systems, McKinley Place NE, Minneapolis, MN, United States), diluted 1:1,000 in Tris-buffered saline (TBS) Tween 20 0.1%, plus 2% BSA and 3% non-fat milk and incubated overnight at 4°C. Membranes were then incubated for 1 h at room temperature with HRP-conjugated anti-mouse and anti-rabbit secondary antibodies, diluted 1:2,000 in TBS Tween 20 0.1% plus 2% BSA and 3% non-fat milk.

Membranes were then visualized by using Clarity Western ECL Substrate (Bio-Rad, Alfred Nobel Drive Hercules, CA, United States), according to the manufacturer’s instructions. Anti-actin antibody was used as control of protein loading.

Densitometry of FasL, Pro-Caspase 8 and c-FLIP was carried out with Fiji ImageJ software. An equal area was selected inside each band, and the mean of gray levels (in a 0–256 scale) was calculated. Data were then normalized to values of background and of control actin band (Pisciotta et al., 2018). Moreover, the expression of FasL in hDPSCs after co-culture with PBMCs was further evaluated by immunofluorescence analysis, as described above. Fas expression was also investigated in hDPSCs cultured alone, through FACS and immunofluorescence analyses.

### Stimulation of hDPSCs With Human FasL Recombinant Protein and Human FasL Inhibitor

Prior to perform stimulation of hDPSCs with human FasL recombinant protein (FasL rc) and human FasL inhibitor (FasL inb) for experimental purposes, the effects on hDPSCs viability and the potential toxicity of different concentrations of FasL rc and FasL inb were evaluated, in order to determine the experimental doses to mimic the exposure of hDPSCs to pre-activated PBMCs.

hDPSCs were seeded at 7,000 cells/cm^2^ in a 96-well plate in α-MEM plus 10% FBS, 2 mM glutamine, 100 units/ml penicillin, and 100 μg/ml streptomycin and cultured upon sub-confluence. Then, culture medium was supplemented with different concentrations of FasL rc (0.1, 0.2, 0.5 ng/ml) and with 0.5 ng/ml FasL rc + 500 ng/ml FasL inb. Cells were kept under stimulation for 24 h. hDPSCs cultured without stimulation were used as controls.

Subsequently, the viability of hDPSCs after FasL rc stimulation was determined by using the standard MTT [3-(4,5-dimethylthiazol-2-yl)-2,5-diphenyltetrazolium bromide] assay. Cells were incubated for 3 h with MTT reagent at 37°C. After incubation, the purple formazan crystals were dissolved in DMSO, at room temperature, then absorbance was measured at OD = 590 nm by using a multiwell plate reader (Thermo Scientific Appliskan, Thermo Fisher Scientific). Besides this, cell proliferation in hDPSCs exposed to different FasL rc concentrations was evaluated by Western Blot analysis of PCNA and densitometry analysis was carried out as described above. To evaluate the expression levels of CCND, CCNA and CCNB, hDPSCs from each experimental group were investigated by real-Time PCR. In particular, cells were homogenized, and total RNA was extracted and purified using the PureLink RNA columns (Thermo Fisher Scientific). cDNA synthesis was performed by using Maxima First Strand cDNA Synthesis Kit with DNase I treatment (Thermo Fisher Scientific). Quantitative real-time PCRs were performed using SYBR Green Master mix (Bio-Rad) on CFX Connect Real-time PCR instrument (Bio-Rad), with the following oligonucleotides: hRPLP0 (F: TACACCTTCCCACTTGCTGA, R: CCATATCCTCGTCCGACTCC) hCCND (F: CATCTACACC GACAACTCCATC, R: TCTGGCATTTTGGAGAGGAAG), hC CNA (F: AATGGAACACTTGCTTCTGAAAG, R: CTTCAAGT AGACTCAGCTCTGC), hCCNB (F: CCTCCCTTTTCAGTCC GC, R: CTCCTGTGTCAATATTCTCCAAATC). Relative quantification was calculated from the ratio between the cycle number (Ct) at which the signal crossed a threshold set within the logarithmic phase of the given gene and that of the reference hRPLP0. Mean values of the duplicate results of three independent experiments for each sample were used as individual data for 2^–ΔΔCt^ statistical analysis (Zordani et al., 2019).

### Expression of von Willebrand Factor in hDPSCs and in Human Dental Pulp

As well established in literature, hDPSCs reside within dental pulp in close proximity of vessels, thus allowing to define them as pericytes (Janebodin et al., 2013). It is well known that pericytes play a key role in maintaining and regulating endothelial cell structure. Moreover, pericytes in a synergistic fashion with endothelial cells may regulate vessel formation and maturation, during angiogenesis process (Bergers and Song, 2005). In light of this evidence and previous findings demonstrating the ability of hDPSCs to support tissue vascularization *in vivo* (Pisciotta et al., 2012; Pisciotta et al., 2015b), the expression of a typical angiogenic marker such as von Willebrand factor was evaluated in hDPSCs when stimulated with FasL rc. To this purpose, hDPSCs were seeded at 10,000 cells/cm^2^ in ultra-low attachment 6-well plates, and cultured in DMEM-F12 containing 2% FBS, 2 mM glutamine, 100 units/ml penicillin, and 100 μg/ml streptomycin in order to obtain 3D floating spheres, as previously described by Pisciotta et al. (2018). When hDPSCs spheres aggregated, 0.5 ng/ml FasL rc and 0.5 ng/ml FasL rc + 500 ng/ml FasL inb, respectively, were added to culture medium for 24 h. Then, culture medium was removed and cells were recovered and attached to slides by using Cytospin (Cytospin^TM^ 4, Thermo Fisher Scientific, Third Avenue Waltham, MA, United States). Briefly, hDPSCs spheres were collected and resuspended in 300 μl of culture medium, then centrifuged at 1,500 rpm for 10 min in Cytospin^TM^ 4 to let them adhere to slides. Subsequently, cells were fixed with 4% PFA in PBS for 20 min at room temperature, then were permeabilized with Triton 0.1% in PBS for 7 min and immunofluorescence analysis of von Willebrand factor (vWf) was carried out by using a rabbit anti-vWf primary antibody (1:100, Merck Millipore, Burlington, MA, United States), as described earlier.

Human DPSCs cultured as floating spheres without stimuli were used as controls.

At the same time, samples of human dental pulp (*n* = 3) were obtained from healthy donors, after obtaining written informed consent, during routine tooth extraction procedures. After extraction, pulps were washed in PBS and fixed in PFA 4% in PBS for 1 h at room temperature. Then, PFA was removed, with the samples being washed in PBS and then processed for paraffin embedding as described by Carnevale et al. (2018). Paraffin-embedded dental pulps were sectioned by a Microm (Microm HM 315) microtome and 6 sections per sample (5 μm thickness) per experimental condition were cut. Half of the sections underwent routine hematoxylin and eosin (H&E) staining, whereas the other sections underwent immunofluorescence and immunohistochemistry analyses against vWf in order to show the localization of vWf ^+^ cells within human dental pulp.

### Evaluation of FasL rc Stimulation on the Activation of Fas/FasL Pathway

In order to investigate the effects triggered by human FasL rc on hDPSCs at different concentrations, hDPSCs were processed for Western Blot analysis, as detailed above. The expression of FasL, Fas, Caspase 8 (Cell Signaling Technology, Trask Lane Danvers, MA, United States), c-FLIP (R&D systems, McKinley Place NE, Minneapolis, MN, United States), FADD (mouse anti-FADD ab; Santa Cruz Biotechnology, Dallas, TX, United States) was evaluated in hDPSCs after exposure to 0.1 ng/ml, 0.5 ng/ml FasL rc and to 0.5 ng/ml FasL rc + 500 ng/ml FasL inb for 24 h. To this regard, hDPSCs exposed to 1 μM Staurosporine were used as positive control of apoptosis. Densitometry analysis was performed as described earlier.

Furthermore, the expression of c-FLIP was also evaluated in hDPSCs after exposure to FasL rc 0.5 ng/ml and to FasL rc 0.5 ng/ml + FasL inb 500 ng/ml, respectively, by immunofluorescence analysis with rabbit anti-c-FLIP ab (Santa Cruz Biotechnology, Dallas, TX, United States). Immunolabeling intensity of c-FLIP expression in both experimental groups was evaluated by pseudocolor analysis: blue to white arrays the colors in a spectrum with blue assigned to a lower value than white (Carnevale et al., 2017).

### Chondrogenic Differentiation of hDPSCs

As mentioned before, hDPSCs hold a wide differentiation potential, although findings in literature report that the ability of these stem cells to reach chondrogenic commitment is debated (Iohara et al., 2006; Zhang et al., 2006). To this purpose, chondrogenic differentiation of STRO-1^+^ /c-Kit^+^ hDPSCs was performed under 3D pellet culture, attempting to mimic the physiological conditions required for articular chondrocyte differentiation (Chen et al., 2015). Briefly, hDPSCs were cultured for 7 and 21 days, respectively, in polypropylene tubes at a density of 5 × 10^5^ cells/tube in chondrogenic medium consisting in DMEM-HG supplemented with 5% FBS, 100 nM dexamethasone and 10 ng/ml TGFβ-3, 10 mM 2P-ascorbic acid (Sigma Aldrich, St. Louis, MO, United States), 1% v/v sodium pyruvate (Life Technologies, Carlsbad, CA, United States), 50 mg/ml ITS premix (BD Biosciences, Franklin Lakes, NJ, United States). To evaluate chondrogenic differentiation, cell pellets from each experimental group were embedded in paraffin and 5 μm thick sections were obtained by microtome. Histological analysis by using Alcian Blue and Masson’s trichrome staining were performed (Pisciotta et al., 2015b; Conserva et al., 2019).

Moreover, chondrogenic commitment was also confirmed by Western Blot analysis of SOX9 (rabbit anti-SOX9 ab; Cell Signaling Technology, Trask Lane Danvers, MA, United States) on hDPSCs after 7 and 21 days of induction. Besides, the expression of Fas, FasL and PCNA was investigated as well to evaluate how Fas/FasL pathway is modulated during the induction of chondrogenic differentiation. Densitometry analysis of SOX9, FasL, Fas and PCNA were carried out as detailed above.

The expression of FasL was further evaluated by immunofluorescence analysis with a rabbit anti-FasL ab (Santa Cruz Biotechnology, Dallas, TX, United States) after 7 and 21 days of chondrogenic differentiation. Undifferentiated hDPSCs were used as controls.

### Chondrogenic Differentiation of hDPSCs With the Addition of FasL rc

In order to further evaluate the role of FasL stimulation on hDPSCs capability to commit toward chondrogenic lineage, FasL rc was added to cells differentiated under pellet culture method. In particular, hDPSCs were seeded in polypropylene tubes at a density of 5 × 10^5^ cells/tube in chondrogenic medium additioned with 0.5 ng/ml Fas rc and 0.5 ng/ml FasL rc + 500 ng/ml FasL inb, respectively, for 24 h. Then, the stimuli were removed and chondrogenic differentiation was carried out for 7 and 21 days. During the 21 days induction time, FasL stimulation was added to chondrogenic medium once a week.

The chondrogenic commitment was evaluated on hDPSCs 3D pellets sections (5 μm thickness), formerly processed for paraffin embedding and microtome cut. Immunofluorescence analysis of SOX9 (Cell Signaling Technology, Trask Lane Danvers, MA, United States) was performed after 7 days of induction and quantification of SOX9^+^ cells was carried out by counting the number of positively labeled cells in 6 randomly selected fields of 15,000 μm^2^ per pellet section (*n* = 6 slides per experimental group). Then, after 21 days of chondrogenic induction, Masson’s trichrome and Alcian Blue stain were carried out in order to evaluate collagen deposition and the production of sulphured acid mucins and GAG, respectively. Values calculated on six slides per experimental group were expressed as mean % area ± standard deviation (SD). All these measurements were conducted by using Fiji ImageJ software. Finally, immunohistochemistry with DAB against Coll-II (mouse anti-Coll-II; Abcam, Cambridge, United Kingdom) was performed at 21 days of differentiation to further analyze the achievement of chondrogenic commitment. Immunohistochemistry was carried out as previously described (Zordani et al., 2019). All the antibodies used in the study are indicated in Table 1.

**TABLE 1:** List of used antibodies.

Name	Host	Source	Dilution	Application
anti-human-CD73-PE-CY7	mouse	BD Biosciences	1:50	FACS
anti-human-CD90-FITC	mouse	BD Biosciences	1:50	FACS
anti-human-CD105-APC	mouse	BD Biosciences	1:50	FACS
anti-human-CD45-PE	mouse	BD Biosciences	1:50	FACS
anti-human-HLA-DR-PE-CY7	mouse	BD Biosciences	1:50	FACS
anti-human-CD34-ECD	mouse	Beckman Coulter	1:50	FACS
anti-human-HLA ABC-FITC	mouse	BD Biosciences	1:50	IF
anti-HLA-DP-DQ-DR-FITC	mouse	BD Biosciences	1:50	IF
anti−FasL	rabbit	Cell Signaling Technology	1:1000	WB
anti−FasL	rabbit	Santa Cruz Biotechnology	1:50	IF
anti-Fas	mouse	Cell Signaling Technology	1:50 1:1000	FACS WB
anti-Caspase 8	mouse	Cell Signaling Technology	1:1000	WB
anti-c-FLIP	rabbit	R&D systems	1:1000	WB
anti-c-FLIP	rabbit	Santa Cruz Biotechnology	1:50	IF
anti-vWf	rabbit	Merck Millipore	1:100	IF
anti-FADD	mouse	Santa Cruz Biotechnology	1:1000	WB
anti-SOX9	rabbit	Cell Signaling Technology	1:1000	WB
anti-Coll-II	mouse	Abcam	1:50	IHC

### Statistical Analysis

All the experiments were performed in triplicate. Data were expressed as mean ± SD. Differences between two experimental conditions were analyzed by paired, Student’s *t*-test. Differences among three or more experimental samples were analyzed by ANOVA followed by Newman–Keuls *post hoc* test (GraphPad Prism Software version 5 Inc., San Diego, CA, United States). In any case, significance was set at *P* < 0.05.

## Results

### Isolation of STRO-1^+^ /c-Kit^+^ Human Dental Pulp Stem Cells and Immunophenotype Characterization

**FIGURE 1:**
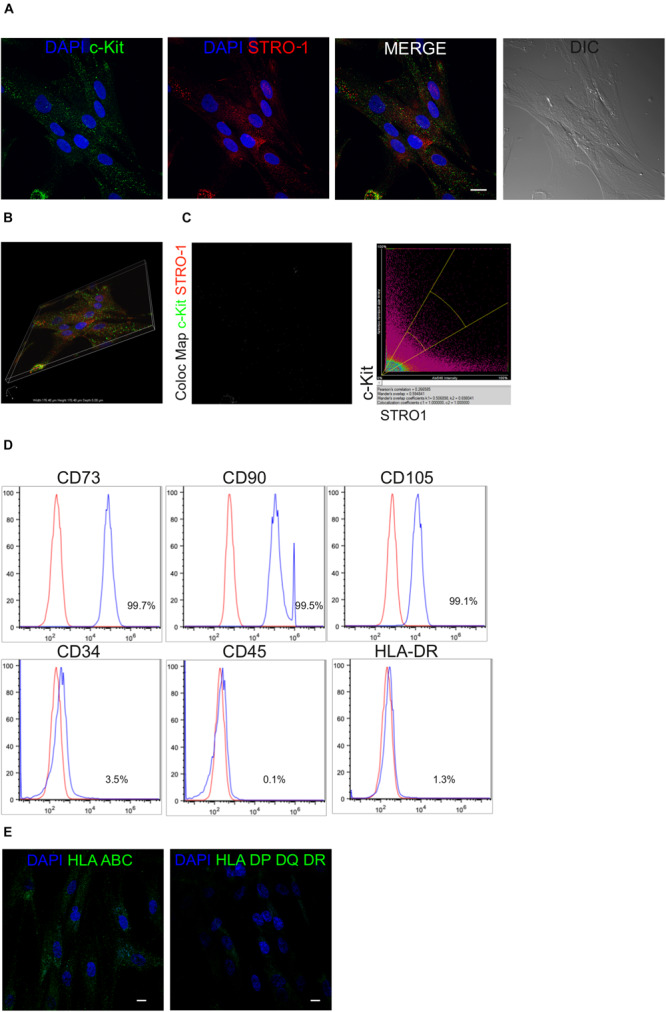
Mesenchymal profile of immune-selected hDPSCs. **(A)** Immunofluorescence analysis on hDPSCs after immune-selection with MACS shows the expression of stemness markers c-Kit (green) and STRO-1 (red). DIC image shows the morphological phenotype of hDPSCs. Nuclei are counterstained with DAPI. Bar = 10 μm. **(B)** Three dimensional reconstruction of optical sections acquired for each fluorescent signal. **(C)** Co-localization binary map of STRO-1 and c-Kit signals is shown. On the right, representative dot plots of co-localization signals are reported. **(D)** Fluorescence-activated cell sorting (FACS) analysis performed on immune-selected hDPSCs. **(E)** Expression of major histocompatibility complex class I (HLA ABC) and class II (HLA DP DQ DR). Bar = 10 μm.

After isolation and expansion of hDPSCs *in vitro*, magnetic immunoselection allowed to obtain a STRO-1^+^/c-Kit^+^ hDPSCs population. The expression of these two surface markers in the selected stem cell population was confirmed by confocal immunofluorescence analysis (Figures 1A,B). Almost all immune-sorted cells were positively labeled against c-Kit and STRO-1 surface antigens, which were distinctly expressed and detected, as shown by the lack of overlapping signals revealed by the colocalization map (Figure 1C). Moreover, immune-phenotypical characterization through FACS analysis revealed that all the typical MSCs markers were expressed by STRO-1^+^/c-Kit^+^ hDPSCs, while being CD45/HLA-DR negative and, only to a lesser extent, CD34 positive (Figure 1D) which is in accordance with previous findings (Pisciotta et al., 2015a). At the same time, confocal immunofluorescence analysis showed the positive labeling against HLA-ABC antigens and the lack of expression of HLA DP-DQ-DR antigens in the selected hDPSCs population (Figure 1E).

### hDPSCs-PBMCs Co-culture: Evaluation of Fas/FasL Pathway

**FIGURE 2:**
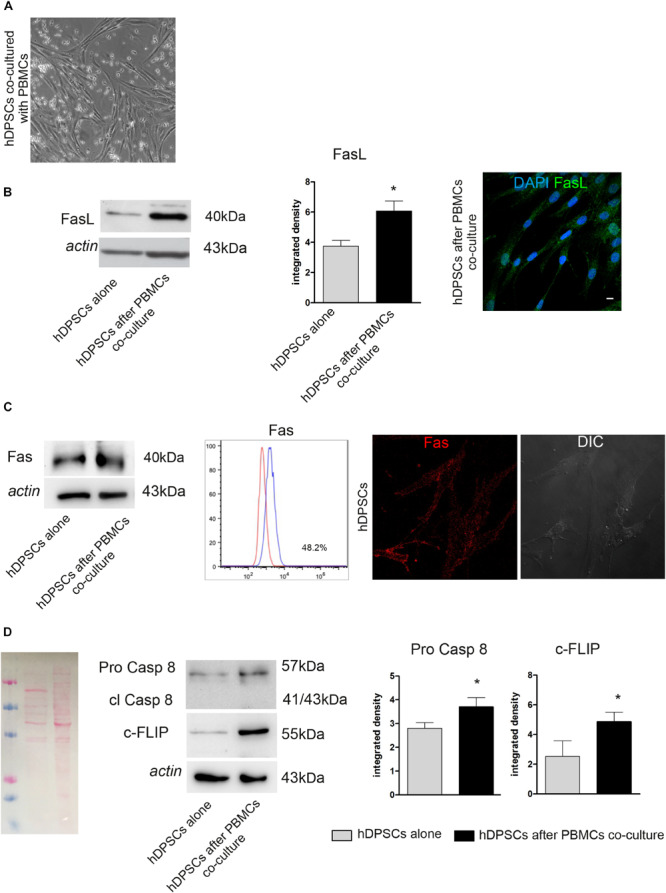
Evaluation of Fas/FasL pathway on both hDPSCs cultured alone and after co-culture with PBMCs. **(A)** Phase contrast image of hDPSCs cultured with PBMCs. **(B)** Western Blot analysis of FasL in both hDPSCs cultured alone and after co-culture with PBMCs. Histograms represent the mean ± SD (*n* = 3) of densitometry of FasL, *P < 0.05 hDPSCs after PBMCs co-culture vs hDPSCs cultured alone. The expression of FasL after co-culture with PBMCs was confirmed by confocal immunofluorescence analysis. Nuclei are counterstained with DAPI. Bar = 10 μm. **(C)** Western Blot analysis of Fas (CD95) in both hDPSCs cultured alone and after co-culture with PBMCs. FACS analysis confirms the positive expression of Fas in hDPSCs cultured alone. The expression of Fas on live hDPSCs cultured alone was also evaluated by Immunofluorescence analysis. DIC Image shows the morphological phenotype of hDPSCs. **(D)** Western blot analysis of pro-caspase 8, cleaved caspase 8 and c-FLIP performed on hDPSCs cultured alone and after co-culture with PBMCs. Actin bands were presented as control of protein loading; histograms represent mean ± SD, *P < 0.05 hDPSCs after PBMCs co-culture vs hDPSCs cultured alone.

After co-culture of hDPSCs and PBMCs for 48 h (Figure 2A), the expression of FasL, Caspase 8 and c-FLIP was evaluated by Western Blot analysis in hDPSCs cultured alone and in hDPSCs after co-culture (Figure 2B). Densitometry analysis showed a significant increase in FasL expression in hDPSCs after co-culture with PBMCs, when compared to hDPSCs cultured alone (^∗^*P* < 0.05 vs hDPSCs alone; Figure 2B). This data was confirmed by immunofluorescence analysis (Figure 2B). Contemporarily, Fas expression was detected in hDPSCs after co-culture with PBMCs, as demonstrated by Western Blot analysis. Interestingly, no statistically significant difference was shown when compared to hDPSCs cultured alone, indeed FACS and immunofluorescence analyses showed that hDPSCs express Fas yet in standard culture conditions (almost 48% of the cells; Figure 2C). Western Blot analysis also allowed to evaluate the expression of Pro-Caspase 8, which revealed an increased expression in hDPSCs after co-culture (^∗^*P* < 0.05 vs hDPSCs alone; Figure 2D). On the other hand, the antibody did not show any labeling against cleaved Caspase 8, therefore demonstrating that activation of the apoptotic cascade did not occur in any of hDPSCs experimental groups. Western Blot analysis also showed an increase in c-FLIP expression in hDPSCs after co-culture, with respect to hDPSCs alone (^∗^*P* < 0.05 vs hDPSCs alone; Figure 2D).

### Stimulation of hDPSCs With Fas-L rc and Fas-L inb: Cell Viability and Proliferation

**FIGURE 3:**
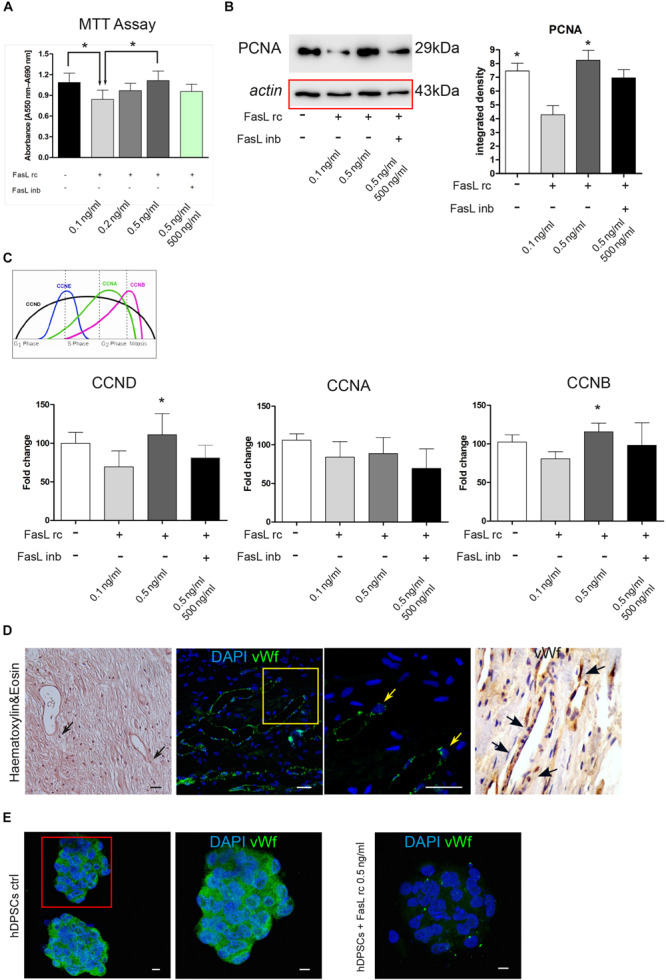
Stimulation of hDPSCs with human recombinant FasL. **(A)** MTT assay was performed on hDPSCs after stimulation with different concentrations of FasL rc. **(B)** Western blot analysis of PCNA in hDPSCs stimulated with FasL rc. Histograms represent the mean ± SD (*n* = 3) of densitometry of PCNA; *P < 0.05 hDPSCs ctrl and hDPSCs treated with FasL rc 0.5 ng/ml vs hDPSCs treated with FasL rc 0.1 ng/ml. **(C)** Real–time PCR analysis showing fold increase of mRNA levels of CCND, CCNA and CCNB in hDPSCs following stimulation with FasL rc. Data represent mean ± SD of fold change obtained from five independent experiments; *P < 0.05 hDPSCs treated with FasL rc 0.5 ng/ml vs hDPSCs treated with FasL rc 0.1 ng/ml. FasL inhibitor was used for FasL rc blocking. **(D)** Representative image of histological section of human dental pulp stained with H&E, magnification 10×. Black arrows indicate the pericytes close to vessels. Immunofluorescence analysis of vWf was performed on paraffin-embedded section of dental pulp. Yellow square indicates the area of high magnification (right side). Yellow arrows highlight vWf positive pericytes. On the right, immunohistochemistry analysis of vWf with black arrows indicating the presence of vWf^+^ cells close to vessels, magnification 20×. Bar = 50 μm. **(E)** Immunofluorescence analysis of vWf was carried out on hDPSCs 3D spheres after treatment with FasL rc 0.5 ng/ml. hDPSCs 3D spheres untreated were used as control. Bar = 10 μm.

Following the stimulation with FasL rc and FasL inb, MTT analysis showed an increase in cell viability at increasing concentrations of FasL rc, indeed, when the highest concentration was used (0.5 ng/ml) cell viability was higher than in the untreated control (Figure 3A). In particular, the untreated control and hDPSCs treated with the highest concentration of FasL rc showed a statistically significant higher viability, compared to hDPSCs treated with the lowest concentration of FasL rc (0.1 ng/ml) (^∗^*P* < 0.05 vs hDPSCs + FasL rc 0.1 ng/ml; Figure 3A). When hDPSCs treated with FasL rc (0.5 ng/ml) were additioned with FasL inb, MTT values were almost reverted to control conditions (Figure 3A). Western Blot analysis of PCNA was also performed on hDPSCs after 24 h of stimulation (Figure 3B). A statistically significant increase in PCNA expression was detected in hDPSCs stimulated with FasL rc 0.5 ng/ml when compared to hDPSCs stimulated with FasL rc + FasL inb (^∗^*P* < 0.05 vs hDPSCs with FasL rc + FasL inb; Figure 3B). The untreated control showed statistically significant higher levels of PCNA, with respect to hDPSCs stimulated with FasL rc 0.1 ng/ml (^∗^*P* < 0.05 vs hDPSCs + FasL rc 0.1 ng/ml; Figure 3B). These data confirmed the results obtained through MTT assay. Likewise, real-Time PCR analysis of mRNA expression of CCND, CCNA and CCNB revealed a statistically significant mRNA fold increase in CCND and CCNB in hDPSCs treated with FasL rc 0.5 ng/ml, thus confirming that the stimulation with FasL rc at its highest concentration triggered a greater proliferation in hDPSCs (^∗^*P* < 0.05 vs hDPSCs ctrl; Figure 3C).

### Expression of von Willebrand Factor in hDPSCs and in Human Dental Pulp

As highlighted in Figure 3D, pericytes can be recognized within human dental pulp close to vessels, by their cell morphology, as shown by H&E stain (black arrows; Figure 3D). Immunofluorescence and immunohistochemistry analyses confirmed the localization *in vivo* of vWf^+^ dental pulp cells adjacent to blood vessels (yellow square and arrows; black arrows; Figure 3D). Immunofluorescence analysis performed on hDPSCs cultured as 3D floating spheres confirmed that cells express the angiogenic marker von Willebrand factor, when cultured under standard expansion conditions. On the contrary, hDPSCs lost the expression of vWf after stimulation with FasL rc at its highest concentration (0.5 ng/ml; Figure 3E).

### Stimulation of hDPSCs With FasL rc and FasL inb: Evaluation of Fas/FasL Pathway

**FIGURE 4:**
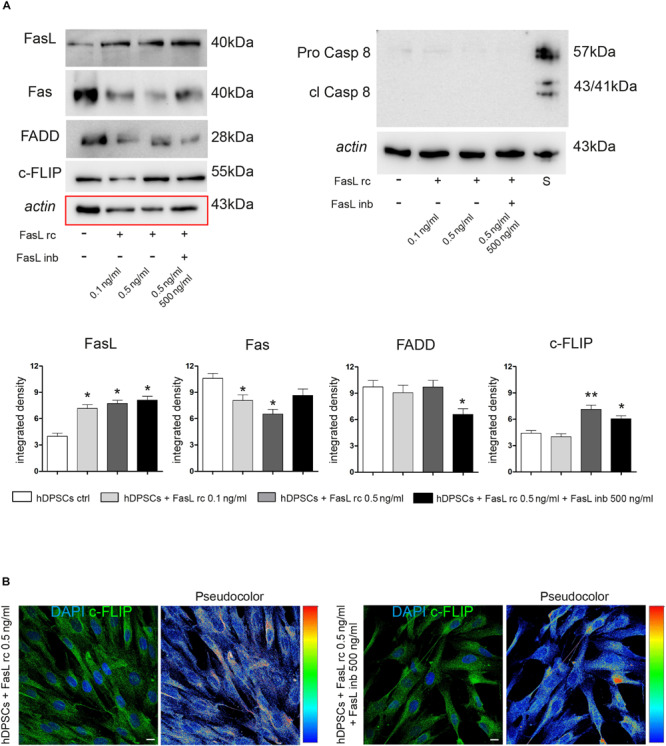
Evaluation of Fas/FasL pathway in hDPSCs following stimulation with FasL rc. **(A)** Western Blot analysis of FasL, Fas, FADD, c-FLIP, pro-caspase 8 and cleaved caspase 8 in hDPSCs after stimulation with FasL rc at different concentrations. hDPSCs treated with 1 μM Staurosporine were used as positive control of cleaved caspase 8. At the bottom, histograms represent mean ± SD (*n* = 3) of densitometry of FasL, Fas, FADD and c-FLIP; *P < 0.05 and **P < 0.01 vs hDPSCs ctrl. **(B)** Confocal immunofluorescence analysis of c-FLIP was performed in hDPSCs stimulated with FasL rc 0.5 ng/ml and with FasL rc 0.5 ng/ml + FasL inb 500 ng/ml. On the right of each immunofluorescent image is reported pseudocolor analysis of c-FLIP staining intensity. Bar = 10 μm.

Following stimulation with FasL rc and FasL inb, Western Blot analyses were carried out to investigate the expression of different proteins related to the Fas/FasL pathway, such as FasL, Fas, FADD, c-FLIP, Pro-Caspase 8 and cleaved Caspase 8 (Figure 4A). As shown by densitometry analyses, there was a statistically significant increase in FasL expression in stimulated hDPSCs compared to untreated control (^∗^*P* < 0.05 vs hDPSCs ctrl).

As far as Fas receptor is concerned, a significant downregulation was observed in hDPSCs stimulated with 0.5 ng/ml FasL rc, compared to the untreated control exhibiting basal levels of Fas (^∗^*P* < 0.05 vs hDPSCs ctrl). Similarly, a statistically significant decrease in Fas expression was observed in hDPSCs stimulated with 0.1 ng/ml FasL rc (^∗^*P* < 0.05 vs hDPSCs ctrl). On the other hand, a slight – although statistically not significant – decrease in Fas expression was revealed in hDPSCs treated with FasL rc + FasL inb, showing values similar to control cells. The expression of FADD was not affected by stimulation, indeed a slight decrease was noticed only when hDPSCs were treated with FasL rc + FasL inb (^∗^*P* < 0.05 vs hDPSCs ctrl). Conversely, a statistically significant augmentation of c-FLIP expression was demonstrated in hDPSCs following stimulation with 0.5 ng/ml FasL rc and the treatment with FasL rc + FasL inb, respectively (^∗∗^*P* < 0.01, ^∗^*P* < 0.05 vs hDPSCs ctrl). As shown by Western Blot analysis, there was no activation of the apoptotic pathway, as cleaved Caspase 8 was not expressed (Figure 4A). Subsequently, confocal immunofluorescence analysis also confirmed the increase in c-FLIP expression in hDPSCs treated with 0.5 ng/ml FasL rc, when compared to unstimulated control (Figure 4B). The pseudocolor analysis highlighted a higher intensity in c-FLIP immunolabeling in hDPSCs stimulated with FasL rc compared to hDPSCs treated with FasL rc + FasL inb (Figure 4B).

### Evaluation of Chondrogenic Differentiation of hDPSCs *in vitro*

**FIGURE 5:**
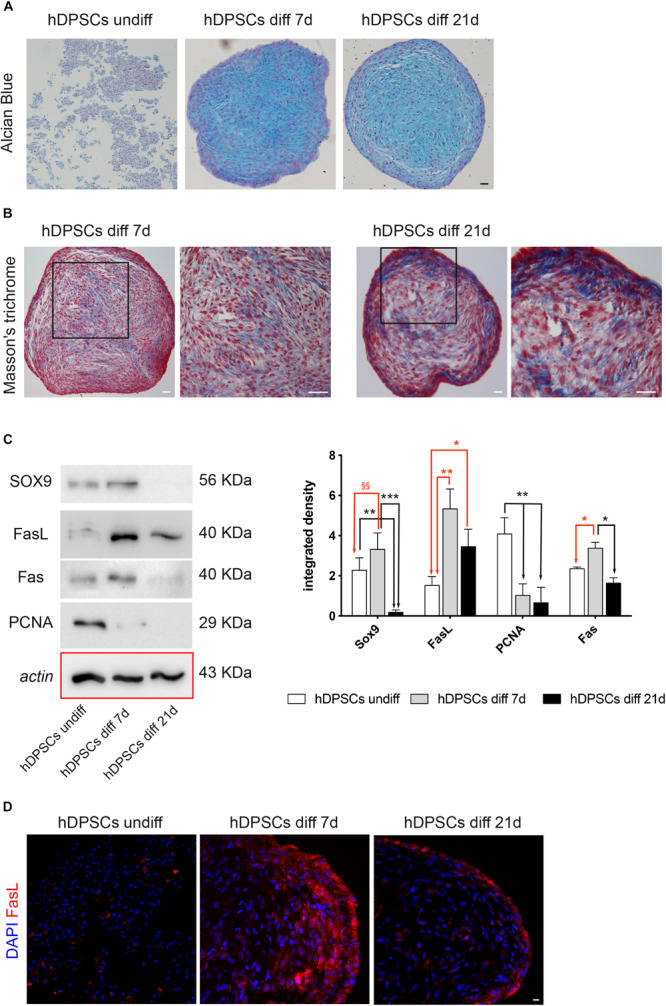
Chondrogenic differentiation of hDPSCs. **(A)** Evaluation of chondrogenic commitment of hDPSCs at 7 and 21 days of induction. Cell pellet sections were stained by Alcian Blue, magnification 10×. Bar = 50 μm. **(B)** Masson’s trichrome staining was carried out to analyze the collagen deposition (blue) in hDPSCs at different time points. Black squares highlight areas of higher magnification on the right side of each image. Bar = 50 μm. **(C)** Western Blot analysis showing expression of SOX9, FasL, Fas and PCNA through the chondrogenic induction of hDPSCs. Histograms represent mean ± SD (*n* = 3) of densitometry of SOX9, FasL, PCNA and Fas. With regard to SOX9: **P < 0.01 hDPSCs undiff vs hDPSCs diff 21-day, ***P < 0.001 hDPSCs 7-day vs hDPSCs diff 21-day; ^§§^*P* < 0.01 hDPSCs diff 7-day vs hDPSCs undiff; with regard to FasL: *P < 0.05 hDPSCs diff 21-day vs hDPSCs undiff; **P < 0.01 hDPSCs diff 7-day vs hDPSCs undiff; with regard to PCNA: **P < 0.01 hDPSCs undiff vs hDPSCs diff 7-day and **P < 0.01 hDPSCs undiff vs hDPSCs diff 21-day; with regard to Fas: *P < 0.05 hDPSCs diff 7-day vs hDPSCs undiff and *P < 0.05 hDPSCs diff 7-day vs hDPSCs diff 21-day. **(D)** Immunofluorescence images showing FasL expression at different times of commitment. Bar = 20 μm.

After *in vitro* expansion, the ability of STRO-1^+^/c-Kit^+^ hDPSCs to differentiate toward the chondrogenic lineage was evaluated. After 7 and 21 days of induction to chondrogenic differentiation, the commitment of hDPSCs, cultured as 3D cell pellets, was investigated through histochemical staining with Alcian Blue and Masson’s trichrome stain. In particular, Alcian blue staining demonstrated the presence of sulphured acid mucins and glycosaminoglycans (GAG) containing carboxylic groups, at 7 and 21 days of induction (Figure 5A). Masson’s trichrome staining highlighted the presence of collagen (blue) after 7 and 21 days of chondrogenic differentiation of hDPSCs. In particular, an increased amount of collagen deposition was revealed after 21 days of induction, with respect to the earlier time of induction (Figure 5B). After 7 and 21 days of culture in chondrogenic medium, the commitment was also evaluated by Western Blot analysis (Figure 5C). In particular, the expression of SOX9, a typical marker of early chondrogenic differentiation, was statistically significant increased after 7 days of induction, while statistically significant decreasing after 21 days of induction, when compared to undifferentiated control (^§§^*P* < 0.01 hDPSCs diff 7-day vs hDPSCs undiff; ^∗∗^*P* < 0.01 hDPSCs diff 21-day vs hDPSCs undiff). Besides, the expression of SOX9 after 21 days of induction was statistically significant lower, when compared to the undifferentiated control and to hDPSCs after 7 days of induction, respectively (^∗∗^*P* < 0.01 vs hDPSCs undiff; ^∗∗∗^*P* < 0.001 vs hDPSCs diff 7-day). Western blot analysis also evaluated the expression of other proteins, such as FasL, Fas and PCNA. The expression of FasL was statistically significant increased after 7 and 21 days of induction (^∗∗^*P* < 0.01 hDPSCs diff 7-day vs hDPSCs undiff, ^∗^*P* < 0.05 hDPSCs diff 21-day vs hDPSCs undiff), while PCNA expression was statistically significant lower at 7 and 21 days of induction, compared to undifferentiated control (^∗∗^*P* < 0.01 vs hDPSCs diff 7-day and hDPSCs diff 21-day). Fas expression showed a statistically significant increase in hDPSCs at 7 days of induction, when compared to undifferentiated hDPSCs and to hDPSCs at 21 days of induction, respectively (^∗^*P* < 0.05 vs hDPSCs undiff, ^∗^*P* < 0.05 vs hDPSCs diff 21-day; Figure 5C). FasL expression was also confirmed by confocal immunofluorescence analysis performed on hDPSCs 3D pellet sections at different times of chondrogenic commitment. The data are in agreement with Western Blot analysis, in fact, the expression of FasL showed an increase after 7 and 21 days of induction to differentiation (Figure 5D).

### Role of FasL Stimulation in Chondrogenic Induction of hDPSCs

**FIGURE 6:**
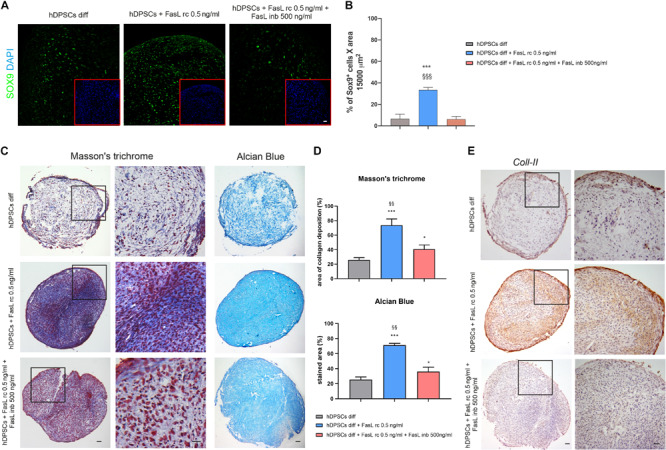
Effects of FasL stimulation on chondrogenic differentiation of hDPSCs. **(A)** Expression of early chondrogenic marker SOX9 was assayed in hDPSCs by confocal immunofluorescence analysis at 7 days of chondrogenic induction supplemented with FasL rc 0.5 ng/ml and with FasL rc 0.5 ng/ml + FasL inb 500 ng/ml, respectively. hDPSCs differentiated without adding FasL stimulation were used as controls (hDPSCs diff). Red squares show nuclei stained with DAPI. Bar = 20 μm. **(B)** Histograms representing the mean percentage of SOX9^+^ cells/15,000 μm^2^ area ± SD. ***P < 0.001 vs hDPSCs diff; ^§§§^*P* < 0.001 vs hDPSCs diff + FasL rc 0.5 ng/ml + FasL inb 500 ng/ml. **(C)** Representative images of Masson’s trichrome and Alcian Blue stained sections from hDPSCs 3D pellet show collagen deposition and the presence of acid mucins and GAG among the three different experimental groups. Black squares indicate areas of higher magnification, reported on the right side. Bar = 50 μm. **(D)** Histograms showing percentage areas of collagen deposition and Alcian Blue stain of 3D pellet sections from each experimental group (*n* = 6). Data are expressed as mean ± SD. *P < 0.05, ***P < 0.001 vs hDPSCs diff; ^§§^*P* < 0.01 vs hDPSCs + FasL rc 0.5 ng/ml + FasL inb 500 ng/ml. **(E)** Immunohistochemistry images showing the expression of late chondrogenic marker Coll-II in differentiated hDPSCs exposed to FasL rc 0.5 ng/ml and FasL rc 0.5 ng/ml + FasL inb 500 ng/ml. hDPSCs differentiated without adding FasL stimulation (hDPSCs diff), were used as controls. Black squares indicate areas of higher magnification, reported on the right side, magnification 10×. Bar = 50 μm.

The induction to chondrogenic differentiation of hDPSCs with the addition of FasL rc and FasL rc + FasL inb was evaluated by confocal immunofluorescence, Masson’s trichrome, Alcian Blue stain and immunohistochemistry analyses. As reported in Figure 6A, SOX9 showed a differential expression among the three experimental groups after 7 days of chondrogenic induction. In particular, as shown in Figure 6B, hDPSCs 3D pellets stimulated with FasL rc 0.5 ng/ml after 7 days of differentiation, revealed a statistically significant higher number of SOX9^+^ cells when compared to hDPSCs 3D pellet differentiated without FasL rc stimulation and to hDPSCs 3D pellet differentiated plus FasL rc 0.5 ng/ml and FasL inb 500 ng/ml, respectively (^∗∗∗^*P* < 0.001 vs hDPSCs diff; ^§§§^*P* < 0.001 vs hDPSCs + FasL rc 0.5 ng/ml + FasL inb 500 ng/ml). These data suggest that the stimulation of hDPSCs 3D pellets with FasL rc + FasL inb triggered a reduction in SOX9 expressing cells to levels resembling hDPSCs diff. As far as later differentiation time is concerned, Masson’s trichrome and Alcian Blue stain highlighted that collagen deposition and sulphured acid mucins and GAG production were greater in hDPSCs differentiated plus the addition of FasL rc stimulation (Figure 6C). As shown in Figure 6D, Masson’s trichrome and Alcian Blue stain quantifications revealed that hDPSCs differentiated plus FasL rc stimulation reached the chondrogenic differentiation to a greater extent, when compared to hDPSCs differentiated without FasL rc stimulation (^∗∗∗^*P* < 0.001 vs hDPSCs diff). When both FasL rc and FasL inb were added, hDPSCs still showed a greater induction with respect to hDPSCs diff (^∗^*P* < 0.05), although resulting in a statistically significant lower induction, when compared with hDPSCs differentiated plus FasL rc stimulation (^§§^*P* < 0.01 vs hDPSCs diff + FasL rc 0.5 ng/ml). Immunohistochemistry analysis of Coll-II further showed that matrix deposition was notably higher in hDPSCs differentiated plus FasL rc stimulation, when compared to hDPSCs diff and hDPSCs diff + FasL rc + FasL inb, confirming the achievement of chondrogenic commitment (Figure 6E).

## Discussion

Human dental pulp stem cells (hDPSCs) represent a heterogeneous cell population enclosed in the loose connective tissue. The immune-selection against the stemness markers c-Kit and STRO-1 allows to obtain a purer stem cell niche with a typical mesenchymal phenotype. It has been widely demonstrated that c-Kit^+^/STRO-1^+^ hDPSCs are able to differentiate *in vitro* toward all the three germ layer derived lineages (Laino et al., 2005; Carnevale et al., 2013) due to their peculiar embryological origin from the neural crest. The multipotency of hDPSCs has been further confirmed by several findings reporting their regeneration potential when applied to different animal models of tissue injuries. In particular, when hDPSCs were studied for the regeneration of bone, muscle and peripheral nerve tissues, not only they committed toward the host tissue cells but also they promoted the tissue healing by triggering angiogenesis (D’Aquino et al., 2007; Pisciotta et al., 2015b; Zordani et al., 2019). To this regard, it is noteworthy that hDPSCs can be localized within dental pulp in the perivascular area, thus being defined as pericytes (Janebodin et al., 2013). Although it has been demonstrated that hDPSCs are able to commit toward the mesenchyme related lineages, the chondrogenic differentiation ability of hDPSCs is still controversial.

It is well known that from an embryological point of view, neural crest derived cells contribute to the formation of the craniofacial district tissues. During the embryological development the poorly represented chondrogenic tissue is immediately replaced by newly synthesized bone tissue supported by neo-angiogenesis. The only portion of cartilage tissue is represented by Meckel’s cartilage. This hyaline cartilage, formed in the mandibular process of the first branchial arch of vertebrate embryos, is not involved in bone formation although supporting the intramembranous ossification (Amano et al., 2010). Based on these considerations it can be argued that neural crest derived stem cells require particular conditions to differentiate toward chondrogenic tissue.

In order to understand chondrogenic potential of hDPSCs, 3D culture pellet mimicking the physiological conditions of chondrocytes differentiation was performed. Data from the early phase of chondrogenic induction revealed high levels of FasL suggesting that it may exert a key role in promoting the early induction of chondrogenic commitment, besides being expressed along the whole differentiation time. Therefore, it may be assumed that Fas/FasL pathway is not only involved in the immunomodulation (Zhao et al., 2012; Riccio et al., 2014; Carnevale et al., 2017). To investigate how Fas/FasL pathway affects hDPSCs biological properties, stem cells expressing high levels of Fas were stimulated with different concentrations of human recombinant FasL protein. This stimulation aimed to mimic the effects of the exposure to inflammatory microenvironment and revealed that, not only undifferentiated hDPSCs avoided apoptosis, but were still proliferating. These findings are in accordance with a previous report showing that Fas, a classically pro-apoptotic molecule, is also able to promote increased cell proliferation in different types of cells including hepatocytes (Budd, 2002). Further studies also demonstrated that high levels expression of c-FLIP counteract the signals of cell death toward a growth signal pathway (Ashany et al., 1999).

Interestingly, at early times of differentiation, an increase in SOX9^+^ hDPSCs was evident, thus suggesting a pro-inductive effect of FasL toward chondrogenic commitment. At later times of differentiation, an augmentation in Coll-II deposition was revealed, demonstrating how FasL is able to play a conductive role by leading the chondrogenic commitment of hDPSCs. These findings confirm previous evidence reporting that FasL expressed by mature chondrocytes exerts a cytoprotective effect (Ryu et al., 2012). At the same time, FasL plays an inhibitory role in the promotion of angiogenesis by inducing Fas mediated apoptosis of vascular endothelial cells, and consequently might avoid the cartilage replacement with bone tissue (Sun et al., 2013). Indeed, as reported the FasL stimulation, added to culture medium, was able to decrease the expression of angiogenic marker and favor more suitable microenvironment for chondrogenic induction.

In conclusion, we can affirm that Fas/FasL pathway (1) not only confers hDPSCs the ability to avoid apoptosis induction when exposed to immune cells but also is primary for the maintenance of proliferation properties of hDPSCs, and (2) is inductive and conductive of chondrogenic commitment by inhibiting the expression of angiogenic marker of neural crest derived pericytes.

## Data Availability Statement

All datasets generated for this study are included in the manuscript files.

## Ethics Statement

The study was conducted in accordance with the recommendations of Comitato Etico Provinciale Azienda Ospedaliero-Universitaria di Modena (Modena, Italy), which provided the approval of the protocol (ref. number 3299/CE; 5 September 2017). All subjects gave written informed consent in compliance with the Declaration of Helsinki.

## Author Contributions

AP, GB, and LB designed and performed the experiments, evaluated the data, and wrote the manuscript. RD and EP performed the experiments and wrote the manuscript. SD performed the hDPSCs characterization experiments and data interpretation. AV and RT performed molecular analysis and provided guidance on the data interpretation. AP and CS performed the experiments, contributed to the data interpretation and edited the manuscript. GC managed the overall project, contributed to the data interpretation, and edited the manuscript.

## Conflict of Interest

The authors declare that the research was conducted in the absence of any commercial or financial relationships that could be construed as a potential conflict of interest.
